# *In vitro* evaluation of shear bond strength of orthodontic stainless steel brackets using transillumination

**DOI:** 10.4317/jced.54751

**Published:** 2018-05-01

**Authors:** Keith R. Dobrin, Prashanti Bollu, Kishore Chaudhry, Karthikeyan Subramani

**Affiliations:** 1 Roseman University of Health Sciences, College of Dental Medicine, Henderson, NV, USA

## Abstract

**Background:**

The objective of this study was to compare the effect of transillumination techniques to conventional light curing on shear bond strength (SBS) and adhesive remnant index (ARI) of orthodontic stainless steel brackets.

**Material and Methods:**

240 extracted human maxillary incisors, canines and premolars were randomly separated into four control and four experimental groups, based on tooth type. Labio-lingual thickness was measured. Control groups were light cured from buccal surface and experimental groups from lingual surface (transillumination) from four directions (mesial-distal, incisal-direct, direct, mesial-distal-incisal). SBS was measured using an Instron machine and ARI evaluated by microscopic inspection.

**Results:**

Mean SBS on maxillary central incisors was lower when cured from lingual side in comparison with buccal side for three light cure directions, but direct cure direction showed nearly equal SBS. Statistical significance was observed for mesial-distal cure direction only. In contrast to central incisors, lateral incisors showed a higher mean SBS when treated from lingual side, for two cure directions (mesial-distal and incisal-direct) with statistical significance observed only for mesial-distal light cure direction. Mean SBS was lower when cured from lingual direction in comparison with buccal direction for all cure directions for canines and premolars. For canines statistical significance was observed for all directions, except incisal-direct; whereas for premolars statistical significance was observed for direct and mesial-distal-incisal directions only.

**Conclusions:**

Transillumination is an effective and clinically acceptable light curing technique for bonding orthodontic stainless steel brackets to maxillary central and lateral incisors. For the other teeth groups (canines and premolars) tested, the mean SBS values, using transillumination light curing fell below the acceptable clinical SBS values, indicating that transillumination is not beneficial in light curing brackets on these teeth.

** Key words:**Orthodontic stainless steel bracket, transillumination, shear bond strength.

## Introduction

Bonding of orthodontic brackets on enamel surface of teeth is a routine procedure in orthodontic practice (1). The procedure requires a composite resin adhesive, with options of setting via chemical or light cure (2). Majority of orthodontists use light cure adhesives in practice (3). Light cure, is also referred as light initiated or light polymerized, and uses light energy for curing. Relative to chemical cure adhesives, light cure adhesives have extended working time, allowing the clinician to place the bracket on a precise location. Additionally, there is easy removal of excess composite before premature setting (2,4-8). Studies have cited higher initial bond strength compared to chemical cure (5,6), as well as reduced risk of contamination due to immediate curing (7). The adhesive sets through polymerization, and should have sufficient radiant intensity, correct wavelength of visible light, and optimal curing time (9).

The prevalence of orthodontic bond failure varies between 6.6% and 17.6% (10-15). Good bond strength, measured as shear bond strength (SBS) is achieved by ensuring that photons reach all layers of composite (16) and completely polymerize it (6). Modified light curing methods have been evaluated in the past, which may be more predictable in effectively curing the composite.

Transillumination is the passage of light through a body area or organ. In relation to orthodontic bonding, this would entail directing light through a tooth to the composite on the bracket base (16,17). Light penetrating the tooth may be more effective than light attempting to traverse the metal bonding pad of the bracket, thereby avoiding competition with the bracket for penetration of the light source (1,6). Composite tends to move towards the light source into the etched enamel rods, which in turn has the potential to increase bond strength (17).

Studies (1,6,8,16,17) have tested transillumination light curing techniques with variable results. Tavas & Watts (1) used transillumination technique with light curing at a 45° angle to the lingual occlusal surface, and concluded that transillumination has the capability of enhanced clinical performance based upon the clinically accepted values (5.88-7.85 MPa) of SBS by Reynolds (18). In 1987, King *et al.* (8) using occlusal and buccal light curing, followed by direct transillumination observed that with increased curing time (threefold) there was adequate SBS to withstand oral forces regardless of the thickness of the teeth tested. Oesterle *et al.* (16) using direct transillumination with higher curing time on extracted human maxillary incisors, concluded that transillumination resulted in SBS which was comparable to conventional curing. In 2013, Kumar *et al.* (17) evaluated maxillary incisors compared to premolars with varying transillumination techniques and determined that the amount of light passing through the tooth was dependent on tooth thickness, contrary to results of King *et al.* (8) Using light emitting diode (LED) transillumination curing light with varying intensities on human premolars, by Hervai *et al.* (6), concluded that lower SBS resulted from transillumination unless there was increased curing time and light intensity. Based upon conflicting results of previous studies, there is a need to explore the use of transillumination as a clinically effective, efficient method to bond orthodontic brackets.

The purpose of this study was to evaluate if different transillumination techniques have clinically sufficient SBS values through light curing stainless steel brackets on extracted human teeth.

## Material and Methods

The samples included 240 extracted human maxillary teeth (40 central incisors, 40 lateral incisors, 80 canines, 80 premolars). Teeth were stored in 1% sodium hypochlorite/distilled water solution (Clorox Co., Oakland, CA/Target Brands Inc., Minneapolis, MN) (19) and changed on a weekly basis. The teeth were individually embedded into a 0.5 x 1.0” PVC pipe (LASCO, Brownsville, TN) filled with Type III Dental Stone (GC America Inc., Alsip, IL). The teeth were oriented so that the enamel surface was parallel to the shearing attachment of Instron Electropuls E1000 testing machine (Illinois Tool Works Inc., Norwood, MA), and perpendicular to the floor. 240 stainless steel brackets were purchased from Dentsply GAC (York, PA).

Enamel surface was pumiced (Henry Schein, Melville, NY) for 5 seconds and rinsed with water.

**Figure 1:**
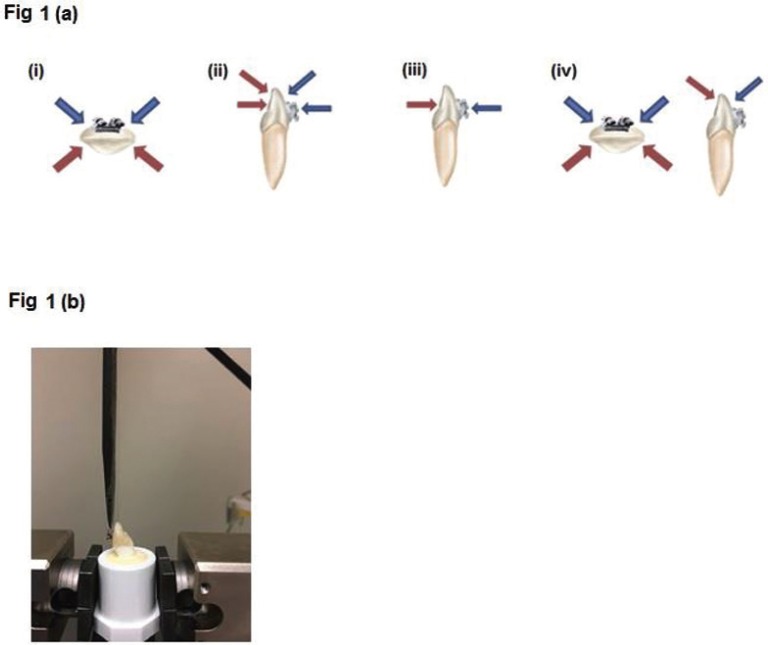
(a) Conventional (Buccal) and Transillumination light curing techniques; (i) mesial-distal, (ii) incisal/direct, (iii) direct, (iv) mesial-distal-incisal. (b) Maxillary central incisor mounted on PVC pipe and positioned in Instron machine. The bracket is bonded on the enamel surface parallel to shearing attachment.

Labio-lingual thickness was measured at the center of the tooth crown using digital caliper (Orthopli Corp., Philadelphia, PA) to the hundredths of a millimeter. Enamel bonding surfaces were etched with unbuffered 35% phosphoric acid (Ultradent Products Inc., South Jordan, UT) for 15 seconds, followed by irrigation with water, then air-dried. A thin layer of bonding primer (Reliance Orthodontic Products, Inc., Itasca, IL) was applied to the enamel surface and air dried, then light cured with an LED light (Ultradent Products Inc., South Jordan, UT) for two seconds. A thin coat of adhesive paste (Reliance Orthodontic Products, Inc., Itasca, IL), was uniformly applied to the bracket base and positioned in the center of the crown, mesio-distally and inciso-gingivally, pressed firmly with 200 grams of force measured using a Dontrix gauge (American Orthodontics, Sheboygan, WI). Any excess composite was removed. The adhesive was light cured (Fig. 1a) as described in  Table 1 for different groups. Incisal and interproximal light curing occurred at a 45° angle from either the buccal or lingual (transillumination) based upon experimental group specification. Following bonding, samples were covered with a moist towel, covered by an opaque tray, and placed into an opaque packing box. The samples were tested for SBS using the Instron at a crosshead speed of 1.0mm/min (20) (Fig. 1b). Bracket surface area (mm2) was provided by Dentsply GAC for each bracket type. Photos of the dislodged orthodontic brackets were taken at 10x magnification and Adhesive Remnant Index (ARI) scores were recorded according to Oz *et al.* (21).

**Table 1:**
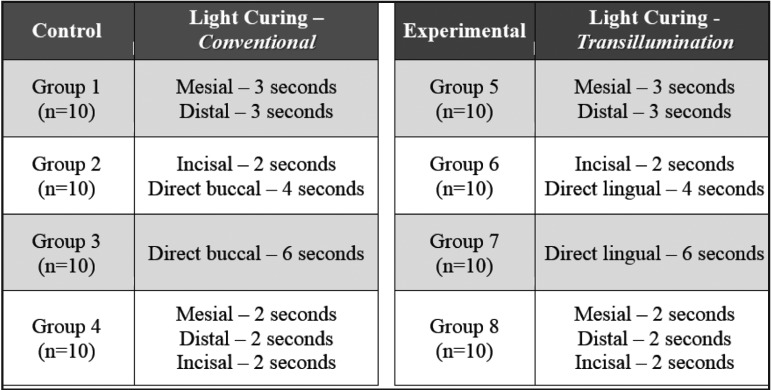
Light Curing specifications by control and experimental groups.

-Statistical Analysis

SBS was measured using the following formula: SBS (MPa) = Force at bracket failure (Newtons) / Surface area of the bracket (mm2)

Data was analyzed using SPSS software version 23.0 (IBM, Armonk, NY). Significance level was set at *p* < .05. The following tests were performed: t-test to compare control and experimental light curing by tooth type, chi-square test to evaluate significance of ARI by light curing method, and correlation test to determine the effect of tooth thickness on SBS for each experimental light curing method by tooth type.

## Results

**Figure 2:**
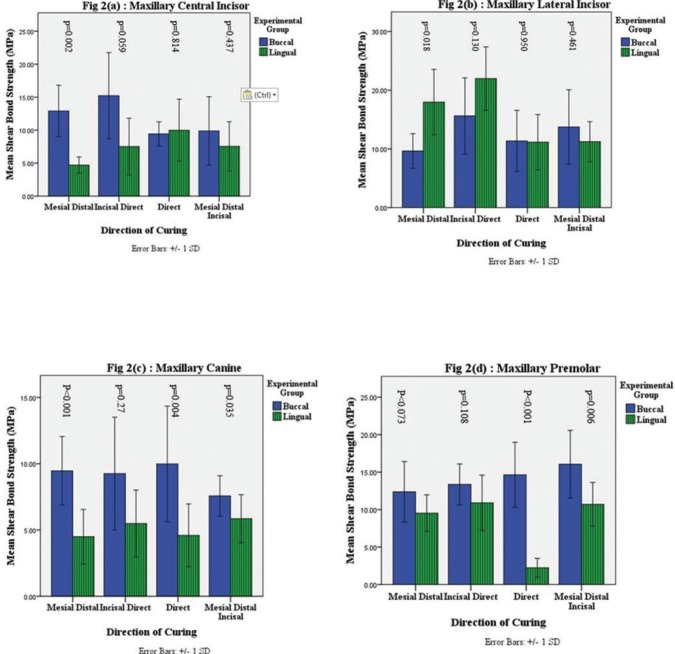
Shear Bond Strength (SBS) according to Buccal-Lingual Light Cure, Categorized by Direction of Light Cure. 2(a) - Maxillary Central Incisor; 2(b) - Lateral Incisor; 2(c) - Maxillary Canine; and 2(d) - Maxillary Premolar. p-value is by t-test.

Mean SBS on maxillary central incisors was lower when cured from lingual side in comparison with buccal side for three light cure directions, but direct cure direction showed nearly equal SBS. Statistical significance was observed for mesial-distal cure direction only (Fig. 2a). In contrast to central incisors, lateral incisors showed a higher mean SBS when treated from lingual side, for two cure directions (mesial-distal and incisal-direct) with statistical significance observed only for mesial-distal light cure direction. Nearly equal but not statistically significant SBS difference was observed for direct and mesial-distal-incisor directions of light cure (Fig. 2b). Mean SBS was lower when cured from lingual direction in comparison with buccal direction for all cure directions for canines and premolars (Fig. 2c, 2d). For canines statistical significance was observed for all directions, except incisal-direct; whereas for premolars statistical significance was observed for direct and mesial-distal-incisal directions only.

**Figure 3:**
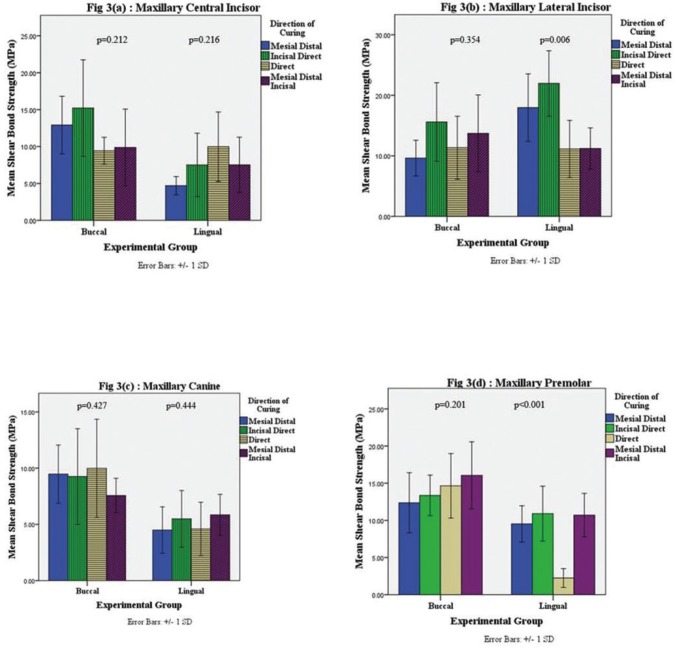
Shear Bond Strength (SBS) according to direction of Light Cure, categorized by Buccal/ Lingual Cure Direction. 3(a) - Maxillary Central Incisor; 3(b) - Lateral Incisor; 3(c) - Maxillary Canine; and 3(d) - Maxillary Premolar. *p*-value is by ANOVA.

**Figure 4:**
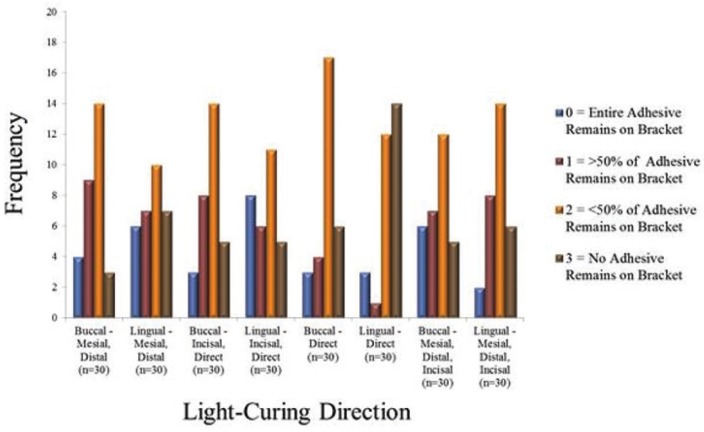
ARI scoring frequency distribution.

Figure 3 shows the SBS for different light cure directions categorized by buccal/ lingual cure, for central incisors (Fig. 3a), lateral incisors (Fig. 3b), canines (Fig. 3c) and premolars (Fig. 3a). Mean SBS was statistically not significant for different light cure groups, except for lingual cure directions for lateral incisors (incisal-direct being statistically significant from incisal-direct and mesial-distal-incisal) and premolars (direct curing being statistically significant from all other 3 cure directions). Figure 4 shows the frequency distribution of ARI scoring. In all groups tested, majority of teeth had an ARI score of 2 (more than 50% of adhesive remaining on the bracket) except direct lingual transillumination group where the majority of teeth had an ARI score of 3 (no adhesive remaining on the bracket).

Correlation between SBS and tooth width was analyzed only for lingual cure group. Teeth with outlier values for SBS and tooth width were removed from this analysis (the results were similar without exclusion as well). As direction of light cure was not observed to be statistically significant, all these groups were clubbed together for analysis ( Table 2). A weak negative correlation was observed for maxillary canines and premolars, but Spearman correlation coefficient was not statistically significant in any of the four tooth categories studied. Moderate positive correlation was observed for lateral incisors which was just short of statistical significance.

**Table 2:**
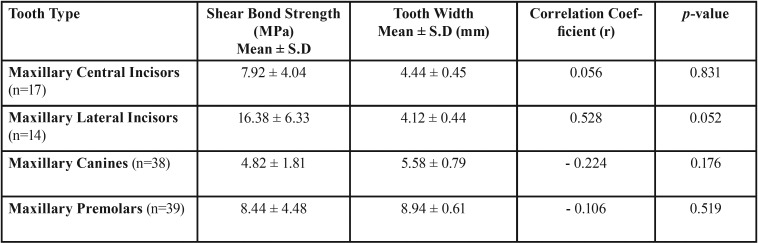
Correlation between Shear Bond Strength and Tooth Width for different tooth types.

SBS data was analyzed for lingual cure group for individual tooth types for light cure direction and tooth width, factorial ANOVA ( Table 3). As a pre-requisite to this test, outlier values for SBS and tooth width were removed from this analysis. No interaction was observed between direction of cure and tooth width, suggesting that their action on SBS was independent of each other. Tooth width was not observed to have statistically significant effect on SBS. Maxillary lateral incisors showed a statistically significant lower mean SBS for direct light cure direction compared to mesial-distal and incisal-direct. In addition, incisal-direct direction was also statistically significantly higher than mesial-distal-incisal cure direction. Maxillary premolars direct light cure showed statistically significantly lower SBS compared to all other cure directions. The results obtained by factorial ANOVA analysis are similar to individual factor analysis (Fig. 2) except that difference in SBS for incisal-direct and mesial-distal-incisal for lateral incisors was slightly short of achieving statistical significance (*p*=0.088).

**Table 3:**
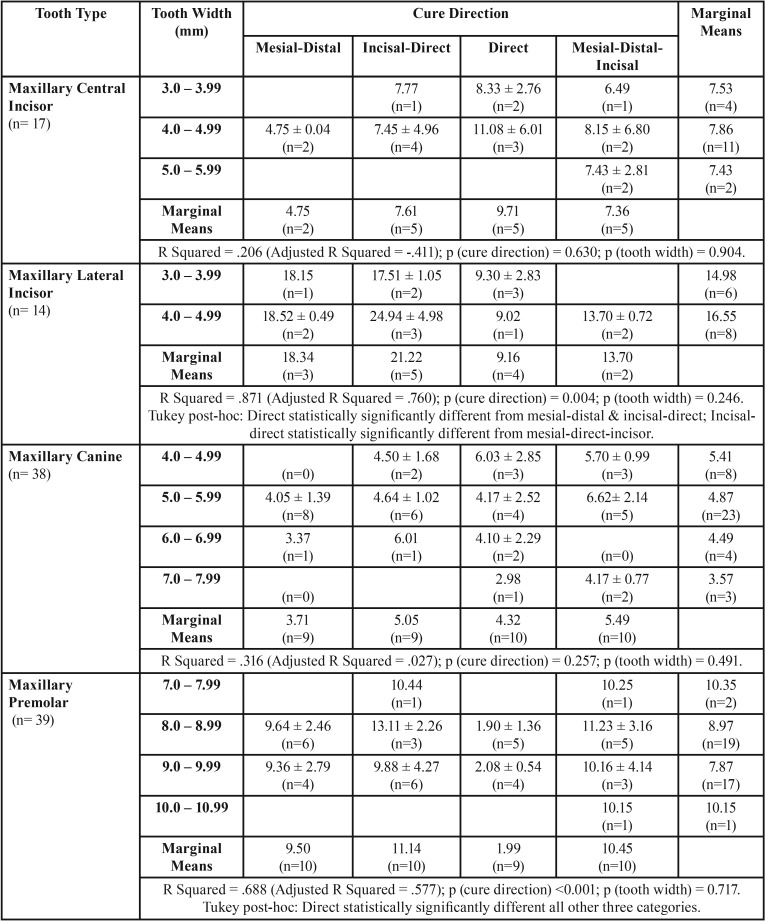
Shear Bond Strength (SBS) (Mean ± SD MPa) for different tooth types in Lingual Cure Group, by Light Cure Direction and Tooth Width. SD = Standard Deviation. *P*-values are from 2-way ANOVA.

## Discussion

Orthodontic bond failure has long been a cause of concern for the clinician, resulting in loss of continuity of patient care, increased treatment length, and decreased profit (22). Creating an ideal bond between the tooth, composite and bracket is of significant importance. However, light may be obstructed from penetrating the metal brackets, resulting in decreased bond strength and bond failure (1,6). Therefore, light must be refracted from within the enamel, dentin, and pulp chamber in order to reach the composite that is blocked by the metal bracket. Numerous studies have evaluated transillumination and conventional light curing techniques in the past (6,7,17,18,24). But these studies have utilized different light sources and different light curing directions and present mixed results. The present study evaluated four conventional curing techniques that have been reported in the literature and from manufacturer recommendations, and compared them to the same four light curing directions using transillumination.

The mean SBS was lower than the clinically acceptable level in maxillary central incisor and canine groups when cured from lingual (transillumination) from a mesial-distal direction and in maxillary canine and premolar groups when cured by direct transillumination. The other curing directions showed higher SBS values than clinically acceptable level in all tooth types. The rationale for this could be that the light has to pass through increased tooth mass when cured from mesial-distal direction on teeth with thicker marginal ridges and through pulp chamber when cured by direct transillumination. These results contradict the conclusions by King *et al.* (8) and Kumar *et al.* (17) that transillumination is a viable and effective technique for light curing orthodontic stainless steel brackets. However, in King *et al.* (8) study, the authors used a halogen light and did not use a control group for comparison and light curing (halogen) included both conventional and transillumination. For Kumar *et al.* (17), a halogen light was similarly used but transillumination was defined as light curing from the occlusal surface for premolars and direct lingual for incisors.

In the present study, SBS values for the specific light curing direction (via transillumination) varied by tooth and should be heavily considered when determining what would be ideal for that individual tooth. The results were similar to Heravi *et al.* (6) who generalized that transillumination resulted in significantly lower SBS values (confirmed in the present study for canines and premolar groups). They stated that one would have to double the curing time and increase the intensity of the light in order to attain acceptable SBS (6), but only premolars were tested with direct transillumination. The present study used an LED with an intensity of 1200 mW/cm2 for six total seconds while they used a maximum of 800 mW/cm2 for 40-80 seconds. Their conclusion, which mirrored Oesterle *et al.*, (16) was that increasing the period of time in light curing with a higher intensity light may have resulted in higher SBS values, yet the latter study also concluded that transillumination alone would adequately light cure labially placed orthodontic brackets (6,16).

In the present study, both the control and experimental groups had predominant ARI scores of 2, with the fracture occurring within the composite. The second highest scoring frequency was the score of 3, interpreted as the fracture occurring at the bracket-composite interface. Therefore, the majority or approximately 2/3rd of scores, regardless of experimental group classification were between 2-3, proving that the strongest bond occurred between the tooth and composite. However, in the direct lingual transillumination group, nearly half of the samples had a score of 3 followed by the score 2. This can be interpreted as less light reaching the adhesive-bracket interface when cured by transillumination. Of the comparative studies which evaluated ARI, only Oesterle *et al.* (16) in the 2001 study utilized transillumination and the same scoring system as the present study. Their results, though, indicated that the site of fracture was predominantly within the composite for control and experimental groups, favoring towards the tooth-composite interface. Kumar *et al.* (17) scored ARI with a different system and the conclusion was that all transillumination groups had scores that reflected more composite was left on the bracket than their corresponding control groups. Two other conventional curing studies (7,23) evaluated ARI and reported a predominance of fractures at the bracket-composite interface.

Tooth thickness and its relationship to SBS has been debated in previous literature, with results ranging from no relationship (8,16), to a negative correlation, meaning thicker teeth have lower SBS values (17). A weak negative correlation was observed for maxillary canines and premolars. Moderate positive correlation was observed for lateral incisors and this effect was contributed by a very high positive correlation coefficient (r) when lateral incisors were treated from incisal-direct cure direction.

One clinical concern when using the transillumination light curing technique is the potential overheating of pulp. It has been determined that increases in pulpal temperature above 42.5°C would produce irreversible damage (24) and therefore contradict the potential usefulness of transillumination. LED curing lights heat the pulp lesser than their halogen counterparts, but the higher output lights with greater intensities might still cause damage (9,25). Extended light curing times and using higher light intensities should be avoided to protect the pulp. The present study utilized low curing time (six total seconds) and moderate light intensity (1200 mW/cm2). Future studies should include a slightly longer curing time to evaluate the effectiveness of transillumination using LED curing lights.

## Conclusions

Transillumination is an effective and clinically acceptable light curing technique for bonding orthodontic stainless steel brackets to maxillary central and lateral incisors. For the other teeth groups (canines and premolars) tested, the mean SBS values, using transillumination light curing fell below the acceptable clinical SBS values, indicating that transillumination is not beneficial in light curing brackets on these teeth.
